# A Novel Closure Technique after Endoscopic Papillectomy Using the Endoscopic Purse-String Suture Device

**DOI:** 10.1055/a-2885-8382

**Published:** 2026-06-11

**Authors:** Yuki Kawasaki, Hisaki Kato, Kazuya Sumi, Jun Ushio, Takayoshi Ito, Noboru Yokoyama, Haruhiro Inoue

**Affiliations:** 1 Digestive Diseases Center 378609 Showa Medical University Koto Toyosu HospitalKotoTokyoJapan

Endoscopic papillectomy (EP) is a therapeutic option for tumors of the duodenal papilla; however, it is associated with adverse events such as bleeding and perforation. 1​ Although closure of the post-EP ulcer after biliary and pancreatic stent placement is generally attempted with clips, achieving complete closure remains challenging. 2​ ​3​ The usefulness of a novel endoscopic purse-string suture technique, “Loop 9,” for closure after gastrointestinal endoscopic submucosal dissection has previously been reported, 4​ ​5​ and a ready-to-use device for this technique has become available ( Fig. 1 ). Using this device, we achieved a tighter closure with less dead space than conventional methods for a post-EP ulcer.

**Fig 1:**
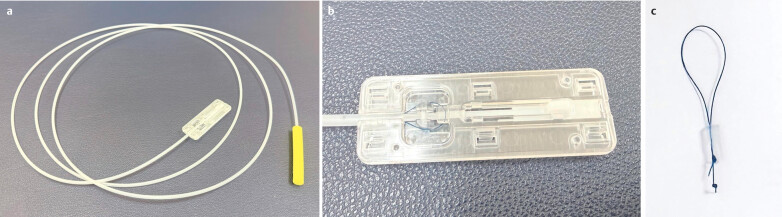
The ready-to-use endoscopic purse-string suture device “Loop 9.” ( **a** ) An overall view of the Loop 9 device. ( **b** ) The Loop 9 system is housed in the distal case. ( **c** ) The Loop 9 loop.

A woman in her 50 s was referred for evaluation of bile duct dilatation. Computed tomography, esophagogastroduodenoscopy, endoscopic ultrasonography, and biopsy revealed an ampullary tumor suitable for EP. After snare papillectomy and specimen retrieval, biliary and pancreatic stents were placed. The loop thread of Loop 9 was positioned over both stents and fixed with clips. Clips were then applied to the anterior and posterior walls on both the distal and proximal sides of the stents ( Fig. 2 ). Finally, the knot at the distal end of Loop 9 was grasped with biopsy forceps and pulled into the sheath; the plastic pledget served as an anchor, allowing the loop to be tightened ( Fig. 2 ).

**Fig. 2:**
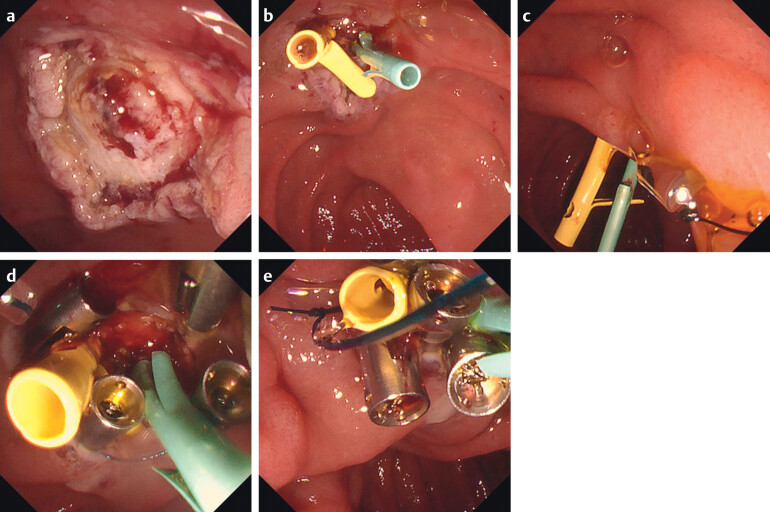
Endoscopic images of Loop 9 closure after endoscopic papillectomy. ( **a** ) Post-papillectomy ulcer after snare resection. ( **b** ) Placement of biliary and pancreatic stents. ( **c** ) Deployment of Loop 9. ( **d** ) Clips were applied to the anterior and posterior walls on both the distal and proximal sides of the biliary and pancreatic stents to fix Loop 9. ( **e** ) Complete closure after tightening Loop 9.

The patient was discharged without adverse events, including delayed bleeding. Two weeks later, the stents were removed, and sufficient healing of the ulcer was confirmed ( Fig. 3 ).

**Fig. 3:**
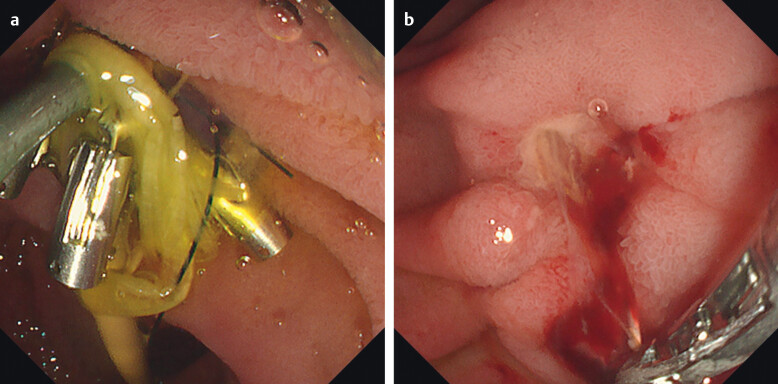
Endoscopic images 2 weeks after endoscopic papillectomy. ( **a** ) The biliary and pancreatic stents, clips, and Loop 9 remained in place. ( **b** ) The bile duct and pancreatic duct orifices after the removal of Loop 9 and the clips.

Loop 9 enabled closure of the proximal side of the stents, which is difficult with conventional clip closure. Suturing around the stents may also prevent stent migration and help reduce bleeding and early stenosis of the bile and pancreatic duct orifices ( Fig. 4 ). This simple and reproducible technique may represent a useful new approach to post-EP closure ( Video 1 ).

**Fig. 4:**
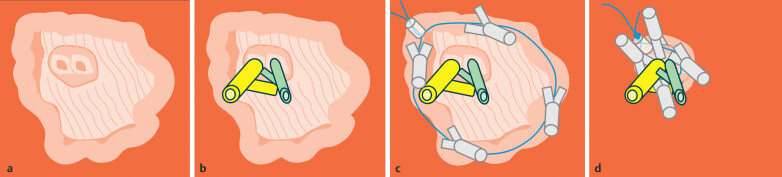
Schematic illustration of Loop 9 closure after endoscopic papillectomy. ( **a** ) Post-papillectomy ulcer after snare resection. ( **b** ) Placement of biliary and pancreatic stents. ( **c** ) Loop 9 was fixed with clips to the anterior and posterior walls on both the distal and proximal sides of the biliary and pancreatic stents. ( **d** ) Closure after tightening Loop 9.

**Video 1** Closure of a post-endoscopic papillectomy ulcer using the endoscopic purse-string suture device.


Endoscopy_UCTN_Code_CPL_1AH_2AL

## Informed Consent

Informed consent was obtained from the patient for the publication of this case report and its accompanying images.

## References

[1] Spadaccini M Fugazza A Frazzoni L Endoscopic papillectomy for neoplastic ampullary lesions: a systematic review with pooled analysis United Eur Gastroenterol J 2020 8 44 51 10.1177/2050640619868367 PMC7006004 32213054

[2] Kagawa K Kubota K Kurita Y Effect of preventive closure of the frenulum after endoscopic papillectomy: a prospective pilot study J Gastroenterol Hepatol 2020 35 374 379 31693767 10.1111/jgh.14922

[3] Fujii Y Matsumoto K Ochi K Clipping closure length is a crucial factor for delayed bleeding after endoscopic papillectomy: a retrospective multicenter cohort study Ther Adv Gastroenterol 2025 1817562848251326450 10.1177/17562848251326450 PMC11915251 40104325

[4] Inoue H Tanabe M Shimamura Y A novel endoscopic purse-string suture technique, “loop 9”, for gastrointestinal defect closure: a pilot study Endoscopy 2022 54 158 162 33472242 10.1055/a-1364-4160

[5] Tanabe M Inoue H Shimamura Y Loop9 closure technique for mucosal defects after colorectal endoscopic submucosal dissection Endosc Int Open 2024 12 E947 E954 39131734 10.1055/a-2362-5617 PMC11309795

